# Mining personalized core traditional Chinese medicine prescriptions for rheumatoid arthritis and elucidating their mechanisms via frequent closed Itemset compression and multilevel network pharmacology

**DOI:** 10.3389/fmolb.2026.1792988

**Published:** 2026-03-23

**Authors:** Xu Chen, Jinlong Yu, Xin Dong, Zhangfan Chen, Jiangshan Tian, Min Zhao, Miao Jiang, Hongtao Guo, Xuezhong Zhou, Lifeng Fa, Yuqiu Li, Lei Zhang

**Affiliations:** 1 Shandong University of Traditional Chinese Medicine, Jinan, China; 2 National Data Center of Traditional Chinese Medicine, China Academy of Chinese Medical Sciences, Beijing, China; 3 Beijing Jiaotong University, Beijing, China; 4 Wangjing Hospital, China Academy of Chinese Medical Sciences, Beijing, China; 5 Institute of Basic Research in Clinical Medicine, China Academy of Chinese Medical Sciences, Beijing, China; 6 The First Affiliated Hospital of Henan University of Traditional Chinese Medicine, Zhengzhou, China

**Keywords:** C-reactive protein, data mining, molecular docking, networkpharmacology, rheumatoid arthritis, traditional Chinese medicine

## Abstract

**Introduction:**

Rheumatoid arthritis (RA) is a complex immune-mediated inflammatory disease involving multiple dysregulated signaling pathways and marked inter-individual heterogeneity in treatment response. In real-world clinical practice in China, traditional Chinese medicine (TCM) is widely used for RA management in the form of multi-herbal prescriptions; however, systematic approaches that link heterogeneous TCM prescription patterns to objective clinical signals and underlying molecular mechanisms remain limited.

**Methods:**

In this study, large-scale inpatient electronic medical records from two tertiary hospitals were analyzed to identify representative TCM prescriptions used for RA treatment. Frequent closed itemset mining combined with compression strategies was applied to extract stable and non-redundant core prescription patterns across different physicians. Retrospective clinical validation was conducted using longitudinal changes in C-reactive protein (CRP) as an objective biomarker of inflammatory activity. Systems pharmacology approaches—including network pharmacology, network topology-based proximity analysis, and molecular docking—were integrated to characterize shared and prescription-specific molecular targets, signaling pathways, and compound-target interaction feasibility.

**Results:**

Five representative core TCM prescriptions were identified. Among 614 eligible patients receiving these prescriptions, all groups exhibited significant post-treatment reductions in CRP levels (p < 0.05), indicating consistent anti-inflammatory signals in real-world settings. Network pharmacology analysis revealed substantial overlap between prescription targets and RA-associated genes (65–115 targets per prescription), with convergent enrichment in key inflammatory pathways, including Toll-like receptor, IL-17, and TNF signaling pathway. Network proximity metrics demonstrated close associations between prescription targets and the RA disease module. Molecular docking further supported the structural plausibility of direct interactions between representative active compounds—such as quercetin and berberine—and core RA-related targets, including TNF-α and PTGS2.

**Discussion:**

This integrative analysis demonstrates that heterogeneous TCM prescriptions used in RA converge on shared inflammatory regulatory networks while retaining prescription-specific mechanistic features. By linking real-world clinical evidence with systems-level and structural analyses, this study provides a reproducible framework for mechanistic interpretation of TCM-based therapeutic heterogeneity and generates testable hypotheses for future prospective and stratified RA studies incorporating standardized clinical outcomes.

## 1 Introduction

Rheumatoid arthritis (RA) is a systemic autoimmune disease characterized by chronic, progressive synovitis and bone destruction, frequently leading to persistent pain, functional disability, and multi-organ involvement, thereby imposing a substantial health and economic burden (Cutolo et al., 2014; Yao et al., 2023). Recent systematic analyses based on the Global Burden of Disease (GBD) study have demonstrated that both the prevalence and disability burden of RA have remained high worldwide and continue to increase (GBD 2021 Rheumatoid Arthritis Collaborators, 2009), posing growing challenges to healthcare resource allocation and long-term disease management (Safiri et al., 2019). From an immunopathological perspective, RA is driven by complex processes including dysregulation of innate and adaptive immunity, amplification of cytokine networks, reciprocal interactions between synovial fibroblast-like cells and immune cells, and involvement of autoimmune mediators such as β2-glycoprotein I (Sorice and Misasi, 2020), which collectively promote chronic inflammation and structural joint damage (McInnes and Schett, 2011; Firestein and McInnes, 2017). Recent integrative reviews have further emphasized that RA is fundamentally a disease of coordinated “immune-stromal” dysregulation, characterized by parallel activation of multiple inflammatory (Smolen et al., 2016a; Smolen et al., 2016b; McInnes and Gravallese, 2021; Radu and Bungau, 2021; Luo et al., 2022), tissue-remodeling, and bone metabolism-related pathways—among which dysregulated Wnt signaling, mediated by key molecules including DKK1, Wnt5a, and β-catenin, serves as a critical bridge linking synovial inflammation to bone erosion (Riitano et al., 2025).

The treatment of RA is multifaceted, typically involving disease-modifying antirheumatic drugs (DMARDs), biologics, and corticosteroids. However, real-world evidence indicates persistent challenges, including heterogeneous treatment responses, long-term adverse effects, and a substantial proportion of patients failing to achieve sustained low disease activity or remission (Burmester and Pope, 2017). The internationally endorsed treat-to-target (T2T) strategy emphasizes dynamic assessment of standardized disease activity measures and timely therapeutic adjustments (Smolen et al., 2010; Smolen et al., 2016b; Smolen et al., 2023), and recent guidelines have further refined treatment algorithms and monitoring strategies across patient populations and disease stages (Fraenkel et al., 2021).

In long-standing clinical practice, TCM treatment of RA is grounded in syndrome differentiation and the use of multi-herb formulae, and has demonstrated potential advantages through multi-component, multi-target, and system-level regulation (Li and Zhang, 2013; Zhang et al., 2019; Li H. et al., 2023; National Clinical Research Center for Dermatologic and Immunologic Diseases et al., 2024; Wen J, et al., 2025), contributing to symptom relief and attenuation of inflammatory responses (Wang et al., 2025). Despite the growing use of TCM, the current evidence for its effectiveness in treating RA remains largely anecdotal, with few systematic studies that link specific herbal prescriptions to measurable biological outcomes. To date, there remains a lack of systematic approaches that leverage large-scale real-world clinical data to extract reproducible core prescription patterns and to establish verifiable evidence chains linking traditional prescriptions to clinical benefit and underlying biological mechanisms.

Existing studies on TCM prescription patterns often employ association rule/frequent itemset mining methods (e.g., the Apriori algorithm) to identify commonly used herb pairs or combinations. However, in high-dimensional, sparse, and highly diverse prescription datasets, such traditional approaches often generate large numbers of redundant patterns, limiting their ability to compactly represent the core co-occurrence structure of prescriptions and reducing reproducibility across physicians and populations (Agrawal and Srikant, 1994). In the field of frequent pattern mining, FP-growth and related candidate-free approaches have highlighted the importance of minimizing redundancy and enhancing interpretability when analyzing large-scale sparse data (Han et al., 2004; Han et al., 2007). Frequent closed itemsets (FCIs), by exploiting closure properties, provide a lossless yet non-redundant representation of all frequent itemsets, substantially improving pattern compactness and interpretability without sacrificing support information (Pasquier et al., 1999; Pei et al., 2000; Liu and Pan, 2004). When combined with compression and novelty-based selection strategies, FCIs can further reduce redundancy and emphasize representative combinations with meaningful incremental information, thereby offering clearer and more actionable inputs for downstream clinical and mechanistic validation (Zhang et al., 2021).

Beyond identifying core herbal combinations, two fundamental scientific questions remain to be addressed: first, whether these core prescriptions correspond to stable and testable clinical benefit signals; and second, through which “compound-target-pathway-disease network” mechanisms such prescriptions may exert therapeutic effects (Hopkins, 2008). With advances in systems biology and computational pharmacology, network pharmacology has emerged as a widely adopted framework for elucidating the mechanisms of multi-component herbal formulas and natural products. By integrating multi-layer information spanning compounds, targets, pathways, and diseases, network pharmacology enables systematic exploration of synergistic interventions in complex diseases and has become a key methodological tool in modern TCM research (Li and Zhang, 2013; Zhang et al., 2019). In recent years, system pharmacology-based studies investigating TCM interventions for immune-inflammatory diseases, including RA, have increased steadily, providing reproducible paradigms for understanding the multi-target and holistic regulatory characteristics of TCM at the systems level (Wang et al., 2017). In parallel, concepts from network medicine and disease module theory propose that disease-associated genes and proteins tend to cluster within molecular interaction networks, thereby offering a theoretical basis for quantifying network distance and proximity between drug targets and disease modules (Barabási et al., 2011; Menche et al., 2015).

At the implementation level, widely used databases and analytical tools—such as TCMSP, SwissTargetPrediction, DAVID, and Cytoscape—support the screening of active compounds, prediction of potential targets, functional enrichment analysis, and network construction (Shannon et al., 2003; Ru et al., 2014; Daina et al., 2019; Sherman et al., 2022). High-quality protein-protein interaction and pathway knowledge bases, including STRING, Reactome, KEGG, and Gene Ontology, provide essential support for PPI network modeling, pathway mapping, and functional annotation (Kanehisa et al., 2021; Gillespie et al., 2022; Aleksander et al., 2023; Szklarczyk et al., 2023). In addition, disease-gene and drug-target resources such as DisGeNET, DrugBank, and ChEMBL supplement disease association evidence and pharmacological target information, enhancing the coverage and traceability of network inference results (Piñero et al., 2020; Knox et al., 2024; Zdrazil et al., 2024). From a statistical and interpretative perspective, beyond conventional over-representation analysis, methods and platforms such as GSEA and g: Profiler are increasingly applied to improve the robustness and interpretability of enrichment results (Subramanian et al., 2005; Kolberg et al., 2023). Furthermore, network proximity or separation metrics between drug targets and disease modules have been extensively adopted in network medicine to quantify system-level drug-disease associations and to compare mechanistic differences across therapeutic strategies (Barabási et al., 2011; Guney et al., 2016; Cheng et al., 2019). At the structural biology level, molecular docking approaches based on the Protein Data Bank (PDB) and engines such as AutoDock Vina and AutoDock4 provide computational support for evaluating the feasibility of direct interactions between key active compounds and core targets (Berman et al., 2000; Morris et al., 2009; Trott and Olson, 2010).

In clinical research and trials, RA treatment efficacy is commonly assessed using composite outcome measures, including the Disease Activity Score in 28 joints (DAS28), ACR20 response criteria, and ACR/EULAR remission definitions, in combination with inflammatory biomarkers such as C-reactive protein (CRP), enabling standardized evaluation of treatment responses (Felson et al., 1995; Prevoo et al., 1995; Felson et al., 2011). Moreover, unified classification and diagnostic criteria for RA (e.g., the ACR/EULAR 2010 classification criteria) form an essential foundation for the comparability of real-world cohorts and the external generalizability of study findings. In this study, CRP was selected as an objective starting point for real-world efficacy validation, facilitating a direct and testable link between prescription mining results and anti-inflammatory signals.

To address these challenges, the present study integrates real-world clinical evidence with systems pharmacology and molecular network analysis. Using large-scale inpatient electronic medical records from two tertiary hospitals, frequent closed itemset mining combined with prescription compression strategies was applied to identify representative core traditional Chinese medicine (TCM) prescriptions used by different physicians for the treatment of rheumatoid arthritis. Retrospective clinical validation was then performed based on longitudinal changes in C-reactive protein (CRP), an internationally accepted biomarker of inflammatory activity, providing objective efficacy signals for the extracted prescriptions in a real-world setting.

**FIGURE 1:**
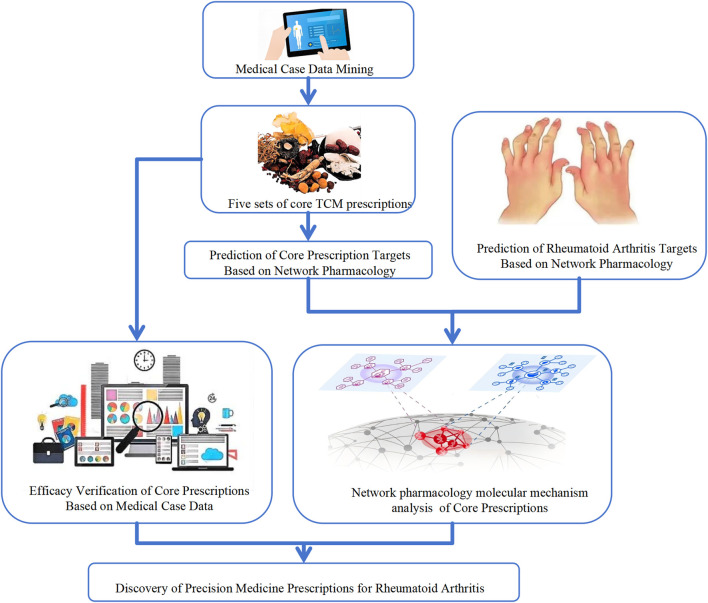
Overall workflow of the study integrating real-world clinical data mining, core prescription identification, efficacy validation, and network pharmacology analysis.

Subsequently, network pharmacology analysis and network topology-based proximity assessment were employed to systematically characterize shared and prescription-specific molecular targets and signaling pathways associated with rheumatoid arthritis, followed by molecular docking to evaluate the structural binding feasibility between representative active compounds and key disease-related targets. Through this integrative, data-driven framework, the study aims to establish a reproducible methodological paradigm for linking real-world clinical signals with molecular mechanisms, thereby bridging clinical practice and systems-level biological interpretation. This approach lays the foundation for future prospective studies incorporating standardized clinical outcomes such as DAS28 and ACR response criteria (Figure 1).

## 2 Materials and methods

### 2.1 Data sources and preprocessing

Clinical data were obtained from the electronic medical record (EMR) systems of hospitalized patients in the Departments of Rheumatology at the First Affiliated Hospital of Henan University of Chinese Medicine and Wangjing Hospital of the China Academy of Chinese Medical Sciences, covering the period from November 2019 to August 2023.

The inclusion criteria were as follows: (1) a primary diagnosis of rheumatoid arthritis (RA) according to the International Classification of Diseases, 11th Revision (ICD-11); (2) a hospital stay of at least 3 days; (3) availability of complete medical record.

The exclusion criteria were: (1) coexistence of severe cardiac, hepatic, or renal dysfunction; (2) pregnancy or lactation; (3) missing clinical data exceeding 20%.

After screening, a total of 4,729 patient records were included, involving prescriptions from multiple attending physicians.

The extracted data comprised: (1) demographic characteristics; (2) clinical manifestations, including chief complaints, history of present illness, specialty examinations, and tongue and pulse characteristics; (3) traditional Chinese medicine (TCM) disease and syndrome pattern diagnoses; (4) laboratory test indicators; and (5) Chinese herbal prescriptions, comprising 450 individual herbal medicines.

The nomenclature of Chinese herbal medicines was standardized in accordance with the Chinese Pharmacopoeia (2025 edition).

### 2.2 Frequent closed itemset mining analysis

#### 2.2.1 Frequent closed itemset mining

Frequent closed itemsets (FCIs) were mined using the CHARM algorithm (Zaki and Hsiao, 2002), a depth-first search-based method that employs a vertical tidset representation and closure checking to directly extract non-redundant closed frequent patterns without candidate generation, making it particularly suitable for high-dimensional and sparse TCM prescription datasets; the minimum support count was set to 1 to ensure comprehensive capture of individualized herbal co-occurrence structures, and the resulting itemsets were further refined using a minimum-coverage-based compression strategy (Zhang et al., 2021) to retain the most representative and information-rich prescription combinations across different physicians. Briefly, the minimum-coverage strategy removes redundant itemsets while retaining a compact subset of representative patterns that can collectively cover the co-occurrence information of the full frequent itemset set.

#### 2.2.2 Screening strategy

For clarity of illustration, the screening process of frequent closed itemsets is exemplified in the following table (Table 1).

**TABLE 1:** Examples of screening results for frequent closed itemsets.

Closed itemset	Number of herbs	Number of prescriptions	Selected	Number of rows	Difference	Difference ratio
DaZao; ShengJiang; YanHuSuo; ChuanXiong; BaiShao	5	108	540	540	540	100.00%
ShaRen; DanShen; FuLing; YanHuSuo; ChuanXiong	5	83	847	415	307	73.98%
ZhiQiao; BaiZhu; ChenPi	3	92	1,123	276	276	100.00%
ZhiGanCao; ChenPi; BaiShao ChuanXiong	4	109	1,322	436	199	45.64%
ZhiGanCao; ChenPi; BaiShao	3	100	1,503	300	181	60.33%
FoShou; BaiShao	2	105	1,638	210	135	64.29%
TaiZiShen; BaiZhu; FuLing	3	66	1,761	198	123	62.12%
NiuXi; DanShen; ChuanXiong	3	83	1,883	249	122	49.00%
HuangQi; BaiZhu	2	78	2,105	156	102	65.38%
NanFangHongDouShan	1	97	2,202	97	97	100.00%

The Chinese medicinal herbs listed in the table above are presented in pinyin for clarity and consistency. The itemsets in this table are ordered according to the screening procedure of the frequent closed itemset mining process (e.g., based on the “Difference Ratio” or the order of extraction), rather than by alphabetical order of herb names. This order reflects the sequential logic of the compression and novelty-based selection strategy described in Section 2.2. The official English and Latin names of the herbs are provided in Supplementary Table S1.

##### 2.2.2.1 Calculation of the difference ratio

$$\text{Difference ratio }\left(\%\right)=\frac{\text{Difference}}{\text{Number}\text{of}\text{rows}}\times 100\%$$

In this context, number of herbs refers to the number of herbal medicines contained in a given frequent closed itemset; number of prescriptions denotes the frequency with which the frequent closed itemset appears across prescriptions; number of rows is defined as the product of the number of herbs and the number of prescriptions. The term selected rows represents the cumulative number of rows selected up to the current frequent closed itemset.

Because subsequently selected frequent closed itemsets may include herbal records that have already been partially covered by previously selected itemsets, the difference represents the number of newly added, non-duplicated rows contributed by the current frequent closed itemset. Specifically, it is calculated as the difference between the current value of selected rows and that of the immediately preceding itemset. For example, in the penultimate itemset shown in the table (NiuXi, DanShen, and ChuanXiong), ChuanXiong had already been partially covered by earlier itemsets containing this herb.

The Chinese medicinal herbs in this study are presented in pinyin. For their official English and Latin names, please refer to Supplementary Table S1.

The difference ratio was used to quantify the novelty of each frequent closed itemset relative to the previously selected set. A difference ratio of 100% indicates that the herbal combination has not been covered by any prior frequent closed itemset, whereas higher values reflect greater novelty and incremental information contribution.

##### 2.2.2.2 Further screening strategy

Although frequent closed itemsets are capable of fully covering prescription patterns, their total number remains large. To facilitate interpretation and downstream analysis, further screening was performed to retain a manageable set of representative frequent closed itemsets according to the following criteria:

1. For frequent closed itemsets with relatively low occurrence frequency, itemsets with high novelty and multiple herbs were retained. Specifically, for itemsets with a number of prescriptions less than 50, only those with a difference ratio equal to 100% and a number of herbs greater than 1 were retained; all others were excluded.
2. For single-herb itemsets (number of herbs = 1), only those with a difference ratio greater than 50% were retained; the remaining single-herb itemsets were removed.
3. Among the remaining frequent closed itemsets, those with low novelty were further excluded. Specifically, itemsets with a difference ratio less than 10% were removed.

This multi-step filtering strategy ensured that the final retained frequent closed itemsets were both representative and information-rich, while minimizing redundancy.

### 2.3 Clinical efficacy validation of core prescriptions

Real-world data (RWD), derived from routine clinical practice, can reflect treatment effectiveness under actual healthcare conditions beyond the constraints of randomized controlled trials, and have been widely applied in evaluations of drug efficacy and safety (Sherman et al., 2016). In the context of chronic diseases and individualized treatment strategies, real-world evidence (RWE) provides an essential complement to conventional clinical trials and plays an irreplaceable role in retrospective study designs (Pei et al., 2000; Zaki and Hsiao, 2002).

After identifying the core prescriptions, retrospective clinical efficacy validation was first conducted to confirm their therapeutic value in real-world settings and to provide an efficacy-based starting point for subsequent mechanistic investigations. The internationally recognized biomarker of inflammatory activity, C-reactive protein (CRP), was selected as the evaluation indicator. CRP levels are significantly correlated with RA disease activity and the extent of bone destruction, and thus serve as an important marker for assessing treatment response (Fang et al., 2020; Pope and Choy, 2021; Witte et al., 2024). Retrospective efficacy analyses were therefore performed for the selected prescriptions.

#### 2.3.1 Selection of core prescriptions

Based on the results obtained from frequent closed itemset mining, core prescriptions were identified using predefined quantitative inclusion criteria to ensure methodological transparency and reproducibility. Specifically, candidate physician-level FCI itemsets were screened according to the following three requirements:

1. Component richness: Each prescription was required to contain at least nine constituent herbs to ensure sufficient structural complexity for downstream systems pharmacology analyses.
2. Clinical recurrence stability: Itemsets were required to demonstrate stable recurrence across patient prescription records, indicating reproducible real-world clinical usage patterns.
3. Information novelty: A difference ratio ≥50% was applied to ensure that each selected itemset provided substantial incremental information relative to previously retained itemsets and to reduce redundancy among prescription patterns.

Itemsets satisfying all three criteria were defined as stable representative prescription patterns. Each retained FCI itemset was directly defined as a core prescription, without any subsequent clustering, merging, or manual modification. Therefore, the number of retained FCI itemsets directly determined the number of final core prescriptions used for downstream efficacy validation and mechanistic analyses.

#### 2.3.2 Clinical indicator-based efficacy analysis of core prescriptions

Among the 4,729 included RA patients, cases treated with the five selected core prescriptions and achieving a medication coverage rate of at least 80% were screened for dynamic CRP analysis. For each patient, CRP measurements obtained before treatment (first test) and after treatment (last test) were collected.

Statistical analyses were performed using SPSS version 26.0. Descriptive statistics were first calculated for CRP levels before and after treatment, including mean values and standard deviations. Paired-samples t tests were then conducted to compare CRP levels before and after treatment. The significance level was set at α = 0.05. A P value <0.05 was considered statistically significant, indicating a meaningful reduction in CRP levels and suggesting that the prescription exerted a significant anti-inflammatory effect in RA patients.

### 2.4 Network pharmacology analysis of core prescriptions

To systematically elucidate the potential biological mechanisms underlying the therapeutic effects of the core prescriptions for rheumatoid arthritis (RA) identified from clinical data, and to explore the modern scientific implications of treatment differentiation under the same disease context, this study performed a network pharmacology analysis of five screened core prescriptions following preliminary clinical efficacy validation based on C-reactive protein (CRP) levels.

System pharmacology studies have demonstrated that analyses of target overlap, pathway enrichment, and network proximity at the network level facilitate a holistic understanding of the interactions between multi-component formulations and complex diseases, thereby reducing the mechanistic bias inherent in single-target assumptions (Wang et al., 2017; Zhang et al., 2019). Accordingly, this study aimed to construct a multi-layered “prescription-active compound-target-signaling pathway” network to systematically predict and compare the key targets, enriched pathways, and topological network properties through which different prescriptions intervene in RA.

Through a systems biology perspective, this approach enables the elucidation of both shared therapeutic mechanisms and prescription-specific intervention tendencies among different formulations, thereby providing computable mechanistic hypotheses and a modern pharmacological interpretation of treatment differentiation and synergistic principles in traditional multi-herbal therapies.

#### 2.4.1 Identification of disease-related targets

RA-related targets were retrieved from public databases, including MalaCards and SymMap, using the keyword “rheumatoid arthritis”. After removing duplicate entries, a curated set of potential RA-associated targets was obtained for subsequent analyses.

#### 2.4.2 Prediction of prescription targets and identification of overlapping targets

Core herbal medicines derived from frequent closed itemsets were screened using the Traditional Chinese Medicine Systems Pharmacology database and analysis platform (TCMSP). The screening criteria for active compounds were oral bioavailability (OB) ≥ 30% and drug-likeness (DL) ≥ 0.18.

Potential targets of the screened active compounds were predicted using SwissTargetPrediction. The predicted prescription-related targets were then intersected with RA-related disease targets to obtain the overlapping targets, which were considered potential therapeutic targets of the core prescriptions.

#### 2.4.3 Functional enrichment analysis

The overlapping target set was imported into the DAVID database for functional enrichment analysis. Kyoto Encyclopedia of Genes and Genomes (KEGG) pathway enrichment and Gene Ontology (GO) enrichment analyses—including biological process (BP), molecular function (MF), and cellular component (CC)—were performed. Pathways and GO terms with p < 0.05 were considered statistically significant.

#### 2.4.4 Network topological property analysis

Network proximity between each core prescription and the RA disease module was evaluated using the following topological metrics:

1. Sab (network separation): values closer to 0 indicate shorter distances between prescription targets and the disease network, suggesting stronger therapeutic relevance;
2. Z-score: negative values indicate significant proximity between prescription targets and the disease module;
3. ASPL (average shortest path length): lower values reflect tighter associations between prescription targets and the RA disease module.

These metrics were calculated based on shortest-path distances between prescription target nodes and RA disease-module nodes in the human protein-protein interaction (PPI) network.

### 2.5 Molecular docking analysis

Molecular docking analysis was conducted as a computational exploration and structural complement to the network pharmacology predictions, aiming to assess the feasibility of direct interactions at the atomic level for direct interactions between active compounds and key RA-related targets, thereby strengthening the structural biology basis of the proposed mechanisms. It should be noted that molecular docking provides structural plausibility of compound-target interactions rather than experimental validation of biological activity, and predicted binding energies should be interpreted with caution.

Network pharmacology analyses predicted potential targets and pathways through which the core prescriptions may exert therapeutic effects. To further verify the capability of active compounds within these prescriptions to directly interact with key RA targets at the structural level, and to provide computational support for the network-based predictions, molecular docking simulations were performed.

#### 2.5.1 Rationale for selection of disease targets

Tumor necrosis factor-α (TNF-α) and prostaglandin-endoperoxide synthase 2 (PTGS2, cyclooxygenase-2, COX-2) were selected as molecular docking receptors due to their central roles in RA-associated inflammation and bone destruction, based on the following considerations.

For clarity, “TNF” in this study refers to the TNF signaling pathway or TNF-related inflammatory signaling context, whereas “TNF-α” specifically denotes the cytokine tumor necrosis factor alpha used in molecular docking and target-level analyses.

##### 2.5.1.1 Role of TNF-α in RA pathogenesis

TNF-α is a key pro-inflammatory cytokine driving synovial inflammation and joint destruction in RA. Its overexpression activates signaling pathways such as NF-κB and MAPK, promotes the release of inflammatory mediators including IL-6 and IL-1β, and induces osteoclast differentiation and activation, ultimately leading to bone erosion (McInnes and Schett, 2011; Schett and Gravallese, 2012; Smolen et al., 2016a; Firestein and McInnes, 2017). Biologic agents targeting TNF-α (e.g., infliximab and etanercept) have become standard therapeutic options for RA, underscoring its importance as a therapeutic target (Feldmann and Maini, 2003; Taylor et al., 2016).

##### 2.5.1.2 Role of PTGS2 in RA inflammation and pain regulation

PTGS2 is a key enzyme in prostaglandin biosynthesis and is highly expressed in RA synovial tissue. Its product, prostaglandin E2 (PGE2), participates in inflammation mediation, angiogenesis, and pain perception (Amin et al., 1997; Crofford, 1997). Nonsteroidal anti-inflammatory drugs (NSAIDs) primarily exert anti-inflammatory and analgesic effects through inhibition of PTGS2, making PTGS2 an important target for symptom control and inflammatory regulation in RA (Vane and Botting, 1998; Ricciotti and FitzGerald, 2011).

#### 2.5.2 Criteria for selection of active compounds

Given the inherent chemical complexity of multi-herb prescriptions, it is computationally and experimentally impractical to validate every constituent in the present study. Therefore, to obtain an initial and exploratory assessment of the potential molecular interactions underlying the network pharmacology predictions, we employed a multi-criteria prioritization strategy to select representative compounds for docking analysis (Park et al., 2023; Fu et al., 2024). It is important to clarify that this approach does not aim to identify all active ingredients or to claim that the selected compounds are the sole drivers of therapeutic efficacy. Instead, the selected compounds serve as exploratory probes to test the structural plausibility of interactions with key RA-related targets, thereby providing a complementary layer of evidence to the network-level associations.

To select representative active compounds from each core prescription for molecular docking validation, the following criteria were applied:

##### 2.5.2.1 Network pharmacology-based prioritization

Active compounds meeting the screening criteria (OB ≥ 30%, DL ≥ 0.18) were first identified using TCMSP in combination with SwissTargetPrediction (Wang et al., 2017; Fu et al., 2024). Compounds showing significant associations with RA-related targets—particularly those involved in the Toll-like receptor (TLR), IL-17, and TNF signaling pathways—were prioritized as candidate ligands.

It is important to acknowledge the inherent limitations of this prioritization strategy. The reliance on databases such as TCMSP and filters like OB ≥ 30% and DL ≥ 0.18 may exclude compounds that are prodrugs, are metabolized into active forms *in vivo*, or exert effects through mechanisms not captured by current cheminformatic parameters. Furthermore, prioritizing compounds with documented bioactivity in the literature may introduce a confirmation bias, potentially overlooking novel or less-studied constituents. Therefore, the selected compounds should be interpreted as representative candidates for hypothesis generation, rather than a definitive or exhaustive list of the active principles within each prescription.

##### 2.5.2.2 Relative importance of compounds within prescriptions

Based on traditional usage and modern pharmacological evidence, compounds considered to play central therapeutic roles within prescriptions were preferentially selected. For example, quercetin is widely present in multiple herbs such as Stellaria dichotoma, Pueraria lobata, and Astragalus membranaceus, and exhibits well-documented anti-inflammatory, antioxidant, and immunomodulatory activities, serving as a common key compound across several prescriptions. Berberine, derived from herbs such as Phellodendron amurense, represents an important active constituent in Prescription 2. Paeoniflorin, a major constituent of Paeonia lactiflora and Paeonia veitchii, contributes to analgesic and anti-inflammatory effects in Prescriptions 2 and 5.

#### 2.5.3 Data preparation

##### 2.5.3.1 Receptor protein preparation

The three-dimensional crystal structures of TNF-α and PTGS2 were obtained from the RCSB Protein Data Bank (PDB). Amino acid sequences and functional annotations were verified using the UniProt database.

##### 2.5.3.2 Ligand preparation

Two-dimensional and three-dimensional structure files (SDF format) of small-molecule ligands with potential anti-RA activity were downloaded from the PubChem database.

#### 2.5.4 Preprocessing for molecular docking

##### 2.5.4.1 Receptor preprocessing

PDBfixer (v2.5.0) was used to remove crystallographic water molecules, buffer ions, and heteroatoms. Missing backbone regions were repaired using homology modeling with Modeller. Hydrogen atoms were added at physiological pH (7.4), and protonation states of key residues were predicted using PropKa to optimize hydrogen-bond networks. The processed structures were converted into PDBQT format using OpenBabel (v3.1.1), with Gasteiger partial charges assigned.

##### 2.5.4.2 Ligand preprocessing

OpenBabel was used to generate initial three-dimensional conformations from two-dimensional ligand structures. Gasteiger charges were assigned, hydrogen atoms were added, and energy minimization was performed using MGLTools. All ligands were finally converted into PDBQT format with defined rotatable bonds and charges.

##### 2.5.4.3 Docking parameter settings

Semi-flexible molecular docking was performed using AutoDock Vina. The docking search space was defined to cover the entire active region of the protein. The docking parameters were set as follows: exhaustiveness = 32 and num_modes = 9. Multiple independent docking runs were performed for each ligand-receptor pair.

#### 2.5.5 Data analysis

For each ligand-receptor complex, the lowest binding energy obtained from AutoDock Vina output was extracted as the binding affinity metric. Protein-ligand interaction analyses were further conducted using the PLIP and Protein Plus platforms to characterize key interactions, including hydrogen bonds, hydrophobic interactions, and π-π stacking, and to generate three-dimensional visualizations of ligand-receptor binding modes.

## 3 Results

### 3.1 Results of frequent closed itemset mining

To compare prescription patterns across different physicians, the results of frequent closed itemset compression were systematically analyzed. Itemsets with a difference ratio below 50% were excluded to reduce redundancy, while itemsets with higher novelty were retained. For each physician, the top ten frequent closed itemsets with the highest representativeness were selected. The above mining results revealed distinct core medication patterns used by different physicians for RA treatment. The retained itemsets for each physician are summarized in Table 2.

**TABLE 2:** Comparison of frequent closed itemset compression results across different physicians.

Physician A	Physician B	Physician C	Physician D
DaZao; ShengJiang; YanHuSuo; ChuanXiong; BaiShao	DangGui; ChuanXiong; BaiShao	**BaiZhi; ChuanWu; WeiLingXian; ZhiGanCao; FangFeng; TaoRen; YiYiRen; ZhiMu; BaiZhu; BaiShao**	**XiXin; GanJiang; YinYangHuo; XianMao; DiFengPi; QianNianJian; GuiZhi; YanHuSuo; YiYiRen; ChuanXiong; FuLing**
ShaRen; DanShen; FuLing; YanHuSuo; ChuanXiong	FuLing; BaiZhu	GuiZhi; DangShen; GanCao; BaiShao	BaiShao; YanHuSuo; YiYiRen; ChuanXiong
ZhiQiao; BaiZhu; ChenPi	GuiZhi; HuangQi	FuLing; HuangQi; DangShen; BaiZhu	CaoWu; ChuanWu; ChuanXiong
ZhiGanCao; ChenPi; BaiShao	ChenPi; GanCao	DangGui; BaiShao	XunGuFeng; QingFengTeng; ZhiMu; RenDongTeng; MuGua; ChuanNiuXi; GuiZhi; BaiShao; YanHuSuo; ChuanXiong; DiFengPi; QianNianJian
FoShou; BaiShao	YanHuSuo; YiYiRen; ChuanXiong	QiangHuo; DuHuo; GanCao	GuiJianYu; ChuanShanLong; HongHua; GanCao; ChuanXiong; DangGui
TaiZiShen; BaiZhu; FuLing	FangFeng; WeiLingXian; BaiShao	ChenPi; GanCao	BaiZhu; FuLing
MaiYa; ShenQu; JiNeiJin	DangShen; HuangQi	JiangCan; MaHuang; ChuanWu; TianNanXing; TaoRen; ZhiGanCao; BaiShao	FangFeng; ZhiGanCao; GuiZhi; BaiShao
HuangQi; BaiZhu	CaoWu; ChuanWu	DuYiWei; BaiZhu	NanFangHongDouShan
NanFangHongDouShan	DuHuo; QiangHuo	SangJiSheng; GuSuiBu	ZhenZhuTouGuCao; AiYe; ShenJinCao; HongHua; CaoWu; ChuanWu; ChuanXiong; SuMu; RuXiang; MoYao; WeiLingXian

Physician E	Physician F	Physician G	Physician hospital
DangGui; ShengDiHuang	DanShen	ChuanXiong; DangGui	DangGui; BaiShao; ChuanXiong
**ChuanShanLong; GuiJianYu; HongHua; YanHuSuo; HuangQi; YiYiRen; DangGui; GanCao; ChuanXiong**	**ChuanShanLong; GuiJianYu; HongHua; YanHuSuo; HuangQi; YiYiRen; DangGui; GanCao; ChuanXiong**	ZhiYuanZhi; LongYanRou; MuXiang; SuanZaoRen; HuangQi; FuLing; BaiZhu; DangShen; ZhiGanCao; DangGui	**YinChaiHu; DaXueTeng; DanZhuYe; QingHao; MuDanPi; BieJia; DiGuPi; ZhiMu; GeGen**
BaiShao; ChuanXiong	DangGui; BaiShao; ChuanXiong	**BaiZhi; ChuanWu; WeiLingXian; FangFeng; TaoRen; ZhiGanCao; ZhiMu; YiYiRen; BaiShao**	FengXianTouGuCao; LuLuTong; ShenJinCao
CaoWu; ChuanWu; GuiZhi YiYiRen	ChuanNiuXi; YiYiRen; GanCao	DiFengPi; QianNianJian; YanHuSuo; ChuanXiong; YiYiRen; BaiShao	ChuanShanLong; BaiZhu
DuHuo; DangGui; ChuanXiong	CaoWu; ChuanWu; GuiZhi; HuangQi	DangGui; BaiZhu; BaiShao	**QinPi; HuZhang; TuFuLing; CheQianZi; ChuanNiuXi; PuGongYing; HuangBai; BiXie; ZeXie; ChiShao; JinYinTeng; WeiLingXian**
ChuanNiuXi; YanHuSuo; YiYiRen; ChuanXiong	FuLing; BaiZhu	GuiZhi; ZhiMu; BaiZhu; BaiShao	FuLing; BaiZhu
GanCao	ShaRen; ChenPi; GanCao	MaHuang; ChuanWu; ZhiGanCao	MangXiao; DaHuang
BaiZhu	QiangHuo; DuHuo	DuZhong; SangJiSheng	LiuJiNu; FengXianTouGuCao
DuZhong; SangJiSheng	DaZao; ShengJiang	MuGua; ChuanXiong; BaiShao	MoHanLian; NvZhenZi
KeTengZi	GuiZhi; BaiShao	HongHua; TaoRen	RuXiang; MoYao
HuangQi	NanFangHongDouShan; DuYiWei	ChenPi; FuLing	MiHouTaoGen; ChuanShanLong

The Chinese medicinal herbs listed in the table above are presented in pinyin for clarity and consistency. The itemsets in this table are ordered according to the screening procedure of the frequent closed itemset mining process (e.g., based on the “Difference Ratio” or the order of extraction), rather than by alphabetical order of herb names. This order reflects the sequential logic of the compression and novelty-based selection strategy described in Section 2.2. Itemsets that met the predefined criteria for core prescription selection are highlighted in bold. The official English and Latin names of the herbs are provided in Supplementary Table S1.

#### 3.1.1 Selection of five core prescriptions

The FCI mining procedure first generated physician-level frequent closed itemsets (Table 2). These itemsets were then filtered according to the predefined inclusion criteria described in Section 2.3.1, ultimately yielding five core prescriptions. Each selected itemset maintained a one-to-one correspondence with a representative prescription pattern without additional clustering or merging, ensuring methodological transparency and reducing subjective selection bias. It should be noted that the physician attribution presented in Table 2 reflects the dominant clinical source in which each FCI pattern was recurrently observed, rather than implying that the prescription was manually defined or selected by an individual physician.

Based on these stable FCI itemsets, five representative prescriptions were defined. Two prescriptions were derived from Wangjing Hospital: Prescription 1 consisting of YinChaiHu; DaXueTeng; DanZhuYe; QingHao; MuDanPi; BieJia; DiGuPi; ZhiMu; and GeGen; and Prescription 2 consisting of QinPi; HuZhang; TuFuLing; CheQianZi; ChuanNiuXi; PuGongYing; HuangBai; BiXie; ZeXie; ChiShao; JinYinTeng; and WeiLingXian. The prescription jointly used by Physician E and Physician F—comprising ChuanShanLong; GuiJianYu; HongHua; YanHuSuo; HuangQi; YiYiRen; DangGui; GanCao; and ChuanXiong—was defined as Prescription 3. The prescription used by Physician D—consisting of XiXin; GanJiang; YinYangHuo; XianMao; DiFengPi; QianNianJian; GuiZhi; YanHuSuo; YiYiRen; ChuanXiong; and FuLing—was defined as Prescription 4. The prescription jointly used by Physician C and Physician G comprising BaiZhi; ChuanWu; WeiLingXian; FangFeng; TaoRen; ZhiGanCao; ZhiMu; YiYiRen; and BaiShao—was defined as Prescription 5.

The selected prescriptions exhibited sufficient herbal composition complexity and recurrence stability to support downstream systems pharmacology analyses, including target aggregation, pathway enrichment, and network topology-based proximity assessment. These five core prescriptions were subsequently subjected to real-world clinical efficacy validation using pre- and post-treatment inflammatory biomarker changes.

### 3.2 Clinical indicator-based efficacy analysis

To evaluate the clinical efficacy of each core prescription in patients with rheumatoid arthritis (RA), C-reactive protein (CRP) was selected as a key biomarker of inflammatory activity. Among the 4,729 included RA patients, cases treated with the five core prescriptions and achieving a medication coverage rate of at least 80% were screened for dynamic CRP analysis. For each patient, CRP measurements obtained before treatment (first test) and after treatment (last test) were collected, and paired-sample t tests were applied to compare pre- and post-treatment values.

#### 3.2.1 Overall efficacy analysis

A total of 614 RA patients met the inclusion criteria for efficacy analysis across the five prescription groups. After treatment, the mean CRP levels in all groups were significantly lower than baseline values, indicating that treatment with the core prescriptions was associated with a significant reduction in systemic inflammatory activity in RA patients.

In Table 3, the observed decreases in CRP levels provide preliminary evidence of consistent anti-inflammatory signals at the biomarker level across all five core prescriptions, thereby establishing an objective clinical anchor for subsequent mechanistic investigations.

**TABLE 3:** Comparison of C-reactive protein (CRP) levels before and after treatment in five core prescriptions.

Prescription group	*n*	CRP before treatment (mg/L)	CRP after treatment (mg/L)	t value	*p* value
Prescription 1	91	78.070 ± 62.613	30.941 ± 37.502	7.83	<0.001
Prescription 2	34	60.965 ± 60.670	28.825 ± 50.387	2.64	0.013
Prescription 3	251	18.559 ± 30.559	11.512 ± 20.278	6.98	<0.001
Prescription 4	119	20.137 ± 30.410	12.807 ± 24.408	7.83	<0.001
Prescription 5	160	27.337 ± 42.654	13.707 ± 29.763	6.98	<0.001

Data are presented as mean ± standard deviation. Paired-sample t tests were performed.

#### 3.2.2 Comparison of efficacy among prescription groups

The statistical results of CRP changes before and after treatment for the five core prescriptions are summarized in Table 3. CRP levels were significantly reduced after treatment in all prescription groups (*p* < 0.05). Among them, Prescription 1 (the Wangjing heat-clearing and dampness-removing formula) exhibited the relatively larger reduction in CRP levels (Table 3).

### 3.3 Network pharmacology results

#### 3.3.1 Identification of disease-related targets

A total of 353 potential rheumatoid arthritis (RA)-related targets were collected from public databases, including MalaCards and SymMap, after removal of duplicate entries.

#### 3.3.2 Prediction of prescription targets and identification of overlapping targets

**FIGURE 2:**
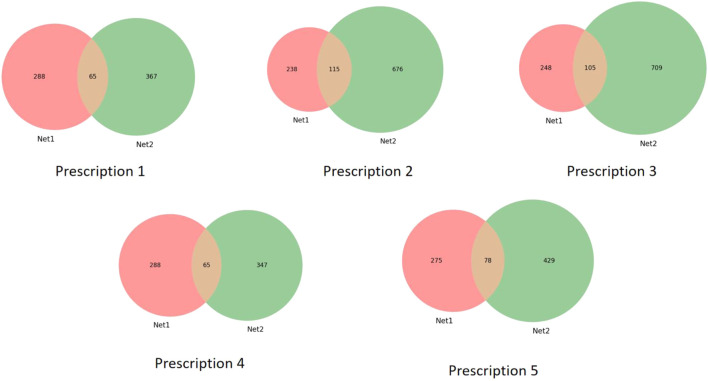
Venn diagram of the five prescription groups.

For each core prescription, potential targets were predicted based on network pharmacology analysis, and overlapping targets with RA-related disease targets were identified (Figure 2).

Prescription 1: A total of 432 prescription-related targets were predicted, of which 65 overlapped with RA-related targets.

Prescription 2: A total of 791 prescription-related targets were predicted, with 115 overlapping targets identified.

Prescription 3: A total of 814 prescription-related targets were predicted, among which 105 overlapped with RA-related targets.

Prescription 4: A total of 412 prescription-related targets were predicted, with 65 overlapping targets identified.

Prescription 5: A total of 507 prescription-related targets were predicted, of which 78 overlapped with RA-related targets.

#### 3.3.3 Functional enrichment analysis

**FIGURE 3:**
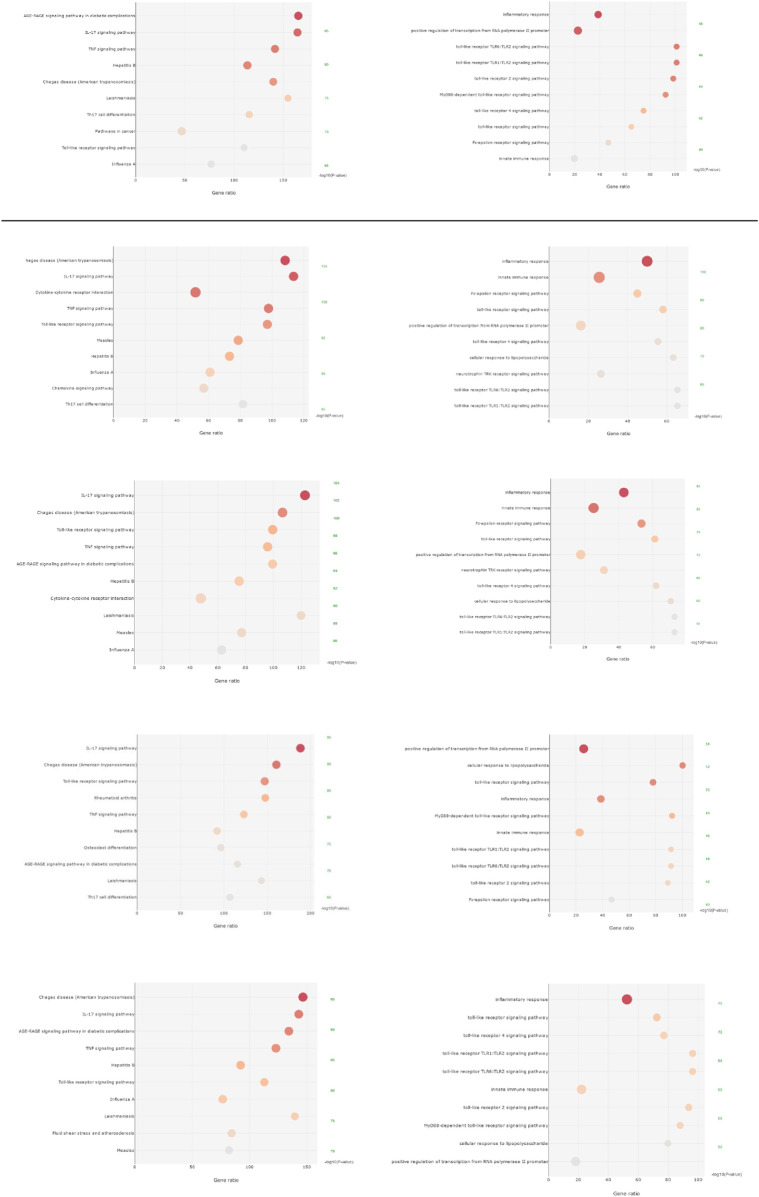
Pathway enrichment analysis of the five core prescription groups. For the enrichment analysis results, rows from top to bottom correspond to Prescriptions 1–5, respectively. From left to right, the first column represents KEGG pathway enrichment analysis, while the second column represents Gene Ontology (GO) enrichment analysis focusing on biological processes (BP). Cellular component (CC) and molecular function (MF) analyses are not shown, as rheumatoid arthritis is primarily associated with dysregulated biological processes related to inflammation and immune regulation.

KEGG enrichment analysis revealed that the overlapping targets of all five core prescriptions were significantly enriched in multiple inflammation- and immunity-related pathways implicated in rheumatoid arthritis. GO biological process enrichment analysis further indicated that these targets were mainly involved in immune response regulation, inflammatory signaling, cytokine-mediated signaling and related biological processes (Figure 3).

#### 3.3.4 Network topological property analysis

Network topology analysis demonstrated that all five prescriptions exhibited Sab values close to 0, relatively small Z-scores, and average shortest path length (ASPL) values approaching 2.8. These results indicate a close network proximity between the targets of each prescription and the rheumatoid arthritis disease module, suggesting close system-level associations (Table 4).

**TABLE 4:** Network topology metrics of the five core prescriptions in relation to the rheumatoid arthritis disease module.

Prescription group	Sab	Z-score	ASPL
Prescription 1	0.012	0.408	2.795
Prescription 2	0.004	−1.268	2.743
Prescription 3	0.012	0.101	2.811
Prescription 4	0.013	0.370	2.826
Prescription 5	0.011	0.278	2.818

Taken together, network topology analysis further supported the core prescriptions and the RA disease module, while enrichment analysis highlighted key pathways potentially modulated by these prescriptions. To further explore the structural feasibility of predicted compound-target interactions, molecular docking analyses were subsequently performed.

### 3.4 Molecular docking results

**FIGURE 4:**
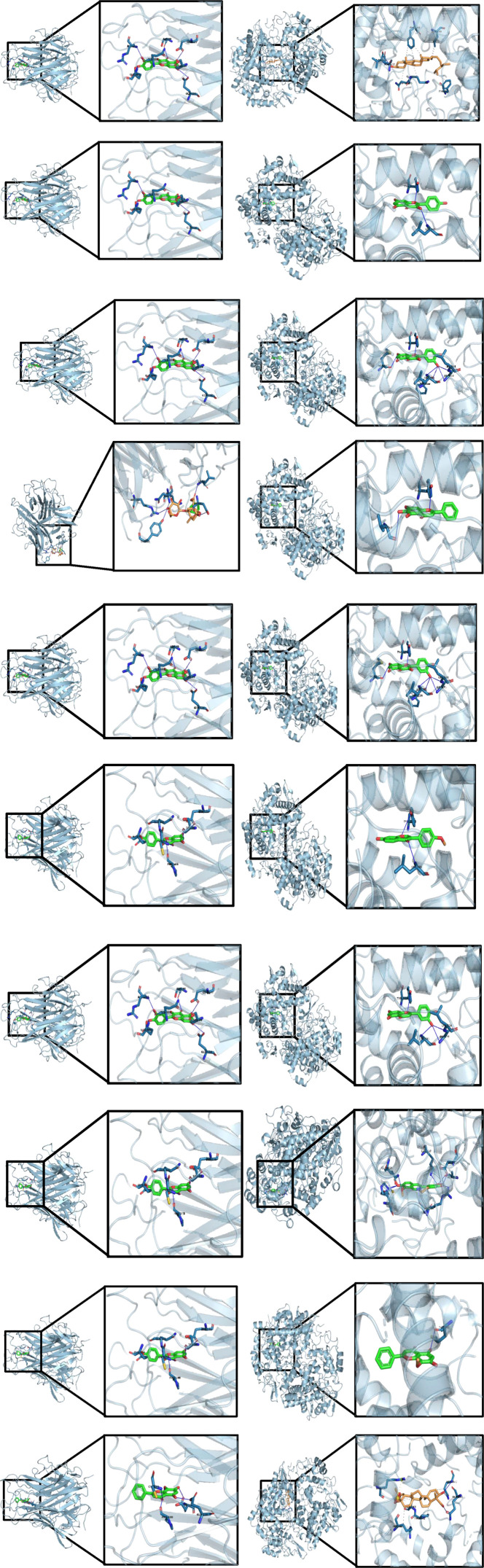
Molecular docking profiles of the five core prescription groups. The docking results are arranged in the same order as listed in Table 5. From top to bottom, rows 1–2 correspond to Prescription 1, rows 3–4 to Prescription 2, rows 5–6 to Prescription 3, rows 7–8 to Prescription 4, and rows 9–10 to Prescription 5. For each prescription, the left panel shows docking with TNF-α and the right panel shows docking with PTGS2.

To evaluate the structural feasibility of potential compound-target interactions predicted by network pharmacology against rheumatoid arthritis at the structural level, TNF-α and PTGS2 were selected as key molecular targets for docking analysis. Major and prescription-specific active compounds identified from the five core prescriptions were docked with these targets. The results showed that multiple natural compounds from each prescription exhibited relatively favorable predicted affinities with both TNF-α and PTGS2, with binding energies generally lower than −7.0 kcal/mol (Table 5). These findings suggest that active compounds derived from the prescriptions may potentially interact with core inflammatory and pain-related targets at the structural level, providing a plausible molecular basis for the observed network-level associations. Previous studies have demonstrated that quercetin can significantly attenuate inflammatory responses in rheumatoid arthritis by inhibiting NF-κB and MAPK signaling pathways (Tang et al., 2022); berberine has been reported to suppress synovial angiogenesis and inflammatory progression in RA (Gao et al., 2024); and kaempferol has been shown to improve RA-associated immune inflammation by regulating Th17 cell differentiation (Li et al., 2024). In addition, various natural flavonoids have been reported to directly target PTGS2 to exert anti-inflammatory and analgesic effects, providing experimental support for the molecular docking results observed in this study (Cui and Jia, 2021) (Figure 4).

**TABLE 5:** Molecular docking results of active compounds from five core prescriptions with TNF-α and PTGS2.

Prescription	Compound (TNF-α)	Binding energy (kcal/mol)	Compound (PTGS2)	Binding energy (kcal/mol)
Prescription 1	Quercetin	−8.632	Beta-sitosterol	−9.626
Prescription 1	Luteolin	−8.484	Kaempferol	−9.664
Prescription 2	Quercetin	−8.632	Quercetin	−9.859
Prescription 2	Paeoniflorin	−6.869	Baicalein	−9.654
Prescription 3	Quercetin	−8.632	Quercetin	−9.859
Prescription 3	Kaempferol	−8.175	Formononetin	−9.167
Prescription 4	Quercetin	−8.632	Luteolin	−9.848
Prescription 4	Kaempferol	−8.175	Berberine	−10.210
Prescription 5	Kaempferol	−8.175	Wogonin	−9.051
Prescription 5	Wogonin	−7.807	Hederagenin	−9.897

As shown in the docking results, multiple active compounds from all five prescriptions were predicted to adopt energetically favorable binding conformations with TNF-α and PTGS2. Notably, quercetin exhibited high binding affinity with TNF-α across Prescriptions 1–4 (−8.632 kcal/mol) and showed strong binding to PTGS2 (−9.859 kcal/mol), suggesting that quercetin may represent a shared candidate compound with favorable structural compatibility across prescriptions. Berberine (Prescription 4) displayed the lowest binding energy with PTGS2 (−10.210 kcal/mol), indicating a potentially prominent role in pain relief and inflammation control. In addition, flavonoid compounds such as kaempferol, luteolin, and baicalein consistently demonstrated favorable binding affinities across multiple prescriptions, further supporting the shared molecular potential of these prescriptions to modulate RA-related inflammatory pathways.

It should be emphasized, however, that these findings do not imply that quercetin and berberine are the exclusive or primary active ingredients responsible for the therapeutic effects. Rather, they serve as representative examples demonstrating that at least some constituents within these complex formulas possess the molecular capacity to directly engage key RA-related targets, providing structural plausibility for the network pharmacology predictions.

In summary, using a set of prioritized representative compounds, the molecular docking analysis predicted energetically favorable binding conformations with TNF-α and PTGS2 across all five core prescriptions. Together, these results offer computational evidence supporting the structural plausibility of interactions between representative active compounds and core RA-associated proteins. It should be emphasized, however, that molecular docking reflects predicted binding feasibility at the structural level rather than experimental validation of biological activity. Therefore, these *in silico* results should be viewed as hypothesis-generating evidence that warrants further experimental validation, rather than definitive proof of *in vivo* efficacy. Together with the network pharmacology enrichment analyses, these findings indicate that although the prescriptions differ in composition, they converge on common and prescription-specific targets through shared or unique active compounds, reflecting the multi-target regulatory characteristics of traditional Chinese medicine.

## 4 Discussion

Based on inpatient electronic medical records from two tertiary hospitals—the First Affiliated Hospital of Henan University of Chinese Medicine and the Rheumatology Department of Wangjing Hospital, China Academy of Chinese Medical Sciences-between November 2019 and August 2023, this study included 4,729 patients diagnosed with rheumatoid arthritis and constructed an integrated evidence chain comprising data mining, clinical efficacy validation (CRP), network pharmacology prediction, and molecular docking verification. RA is a systemic autoimmune disease characterized by chronic synovitis, immune dysregulation and bone erosion, involving complex inflammatory networks such as TNF signaling, IL-6 signaling, IL-17 signaling, Toll-like receptor (TLR)-mediated innate immune activation, and osteoclast differentiation pathways. Therefore, an integrative framework combining clinical signals with mechanistic pathways is particularly suitable for capturing the complex system features of RA (McInnes and Schett, 2011; Smolen et al., 2016a; Firestein and McInnes, 2017).

After the stable co-occurrence patterns and anti-inflammatory efficacy of the five core prescriptions had been presented in the Results section, this Discussion further addresses three key aspects: (1) the role of frequent closed itemset compression and difference-ratio-based screening in improving reproducibility and interpretability of prescription extraction in highly heterogeneous real-world settings; (2) the provision of testable clinical support for mining results through before-after comparisons of the objective biomarker CRP; (3) the elucidation of shared and prescription-specific mechanisms from target-pathway-disease module perspectives, supplemented by molecular docking evidence for structural feasibility of key interactions.

### 4.1 Refinement of core prescription combinations

Real-world clinical prescriptions are characterized by substantial inter-individual variability and physician-specific practice patterns. Reliance solely on simple frequency statistics may lead to redundancy accumulation and threshold-dependent bias, thereby undermining the stability of downstream mechanistic inference. To address this issue, the present study adopted a frequent closed itemset (FCI) compression strategy to perform information-preserving dimensionality reduction of prescription co-occurrence structures, enabling extraction of core combinations with enhanced interpretability and reproducibility. FCIs can be regarded as a non-redundant representation of frequent patterns, substantially reducing duplicate itemsets without loss of frequency information and thereby improving readability and transferability of mining results (Pei et al., 2000; Zaki and Hsiao, 2002).

Specifically, CHARM algorithm was used for FCI mining with the minimum support count set to 1, ensuring comprehensive capture of potential prescription combinations. Subsequently, a minimum-coverage-based compression method was applied to optimize the results, markedly reducing redundancy while retaining key compatibility structures. Apriori-based frameworks remain classical and widely used approaches for extracting co-occurrence rules in medical and prescription data (Agrawal and Srikant, 1994).

To further avoid the oversimplified assumption that “high frequency equals importance”, a difference ratio was introduced to quantify novelty (difference ratio = difference/rows × 100%), where “difference” represents newly added, non-overlapping coverage relative to previously selected itemsets. The screening rules ensured that retained itemsets emphasized incremental information contribution rather than repetitive co-occurrence, providing cleaner and more structured inputs for subsequent clinical and mechanistic validation. Similar observations have been reported in previous real-world prescription studies, highlighting the limitations of pure frequency-based methods and the necessity of structural compression or rule-based filtering to enhance interpretability and clinical utility (Huang et al., 2022; Li X. et al., 2023).

On this basis, five core prescriptions were selected for efficacy and mechanistic validation: Prescriptions 1 and 2 represented two major formulations from Wangjing Hospital; Prescription 3 corresponded to the representative formulation of Physician E and Physician F to that of Physician D and Prescription 5 to that of Physician C and Physician G. The selection emphasized prescriptions directly targeting RA disease activity, thereby enabling construction of a more focused efficacy-mechanism evidence chain. Given that TCM treatment of RA is often driven by syndrome-based individualized strategies, extracting reproducible core combinations from real-world practice and subjecting them to systematic validation aligns with the international paradigm of translating empirical practice into evidence-based frameworks (Pan et al., 2019).

### 4.2 CRP-based clinical efficacy signals

After identifying interpretable core prescriptions, this study further examined whether these combinations exhibited stable anti-inflammatory directional benefits in real-world clinical practice, thereby distinguishing statistical co-occurrence patterns from verifiable clinical signals. C-reactive protein (CRP), an internationally recognized biomarker of inflammatory activity, was selected to establish a testable link between the mined prescriptions and clinical response. CRP is widely used in RA disease activity assessment and is incorporated into composite indices such as DAS28-CRP; however, it is also influenced by infection, obesity, liver function, and inter-individual variability. Accordingly, in real-world settings, CRP should be interpreted as a directional inflammatory indicator rather than a standalone efficacy endpoint (Orr et al., 2018). Notably, because this study was based on retrospective EMR data, standardized composite disease activity indices (e.g., DAS28) were not routinely documented, and ESR testing was inconsistently performed across patients, resulting in substantial missingness that precluded reliable comparative analysis. In routine inpatient practice, laboratory markers such as CRP are generally recorded more consistently than structured joint assessments or longitudinal imaging follow-up. Therefore, the clinical efficacy of the mined prescriptions cannot be comprehensively evaluated within standardized RA outcome frameworks, and the CRP-based findings should be interpreted cautiously as biomarker-level anti-inflammatory signals rather than definitive evidence of remission or structural improvement.

Among the 4,729 RA patients, 614 cases receiving one of the five core prescriptions with medication coverage ≥80% were included for dynamic CRP analysis. Paired t-tests demonstrated significant post-treatment reductions in CRP across all five prescription groups (P < 0.05), indicating consistent anti-inflammatory directional signals in real-world inpatient settings. Prescription 1 exhibited the relatively larger CRP reduction, providing a preliminary clinical clue for subsequent exploration of inter-prescription effect heterogeneity. This approach is consistent with international RA management strategies emphasizing dynamic evaluation using objective and quantifiable disease activity measures within treat-to-target frameworks (Smolen et al., 2016b).

It should be noted that differences in CRP reduction magnitude among prescription groups may reflect distinct pathway emphases, as well as confounding factors such as baseline inflammatory burden, concomitant medications, comorbidity profiles, and inpatient treatment pathways. Given the retrospective real-world design, these differences are better interpreted as indicators of potential heterogeneity and prescription-phenotype matching opportunities rather than evidence of absolute superiority of one prescription over another. Such an interpretation is consistent with current trends toward precision stratification and heterogeneity-aware management in RA (McInnes and Schett, 2011; Smolen et al., 2016a; Firestein and McInnes, 2017).

### 4.3 Mechanistic interpretation: Shared axes and prescription-specific features

Following the observation of consistent anti-inflammatory signals at the clinical level, this study further investigated the potential mechanistic basis underlying these effects from the perspectives of targets and pathways, while exploring biological factors that may contribute to differences in the magnitude of therapeutic responses among prescriptions. Specifically, by integrating network pharmacology with network topology-based proximity assessment, we systematically associated “prescription-compound-target-pathway-disease module” relationships and supplemented these findings with molecular docking analyses to evaluate the structural feasibility of direct interactions between key compounds and core rheumatoid arthritis (RA) targets.

RA is characterized by a complex systemic inflammatory network involving multiple cell types, mediators, and signaling pathways. Accordingly, interpreting the effects of multi-herbal formulations from a multi-target and multi-pathway perspective is theoretically well aligned with the pathophysiological features of RA (McInnes and Schett, 2011; Smolen et al., 2016a; Firestein and McInnes, 2017).

#### 4.3.1 Network-level associations between prescriptions and the RA disease module

From the disease perspective, a total of 353 RA-associated targets were collected. Target prediction and intersection analyses revealed that Prescription 1 contained 432 predicted targets with 65 overlapping RA targets; Prescription 2 contained 791 predicted targets with 115 overlaps; Prescription 3 contained 814 predicted targets with 105 overlaps; Prescription 4 contained 412 predicted targets with 65 overlaps; and Prescription 5 contained 507 predicted targets with 78 overlaps. These results indicate that all five prescriptions share substantial target intersections (65–115 targets) with the RA disease module, providing a target-level basis for their observed anti-inflammatory effects.

Network topology-based proximity metrics further supported this association. All prescriptions exhibited Sab values close to zero (0.004–0.013) and relatively low average shortest path length (ASPL) values (2.743–2.826), suggesting close proximity between prescription targets and the RA disease module and efficient network signal propagation. Notably, Prescription 2 showed a markedly negative Z-score (−1.268), whereas the remaining prescriptions displayed small positive Z-scores (0.101–0.408). This pattern suggests that Prescription 2 may possess a stronger degree of statistically significant network proximity, while the others may reflect a more distributed multi-target synergistic intervention pattern. Collectively, these metrics indicate that all five prescriptions show measurable topological proximity to the RA disease module at the systems level.

#### 4.3.2 Shared inflammatory pathways: A common anti-inflammatory axis

Pathway enrichment analysis revealed that all five prescriptions were significantly enriched in core RA-related inflammatory pathways, including the IL-17 signaling pathway, TNF signaling pathway, and Toll-like receptor (TLR) signaling pathway. IL-17 signaling pathway and TNF signaling pathway are known to synergistically amplify inflammatory responses in fibroblast-like synoviocytes, macrophages, and neutrophils, promoting the expression of chemokines, matrix metalloproteinases, and inflammatory mediators. Meanwhile, the TLR-MyD88-NF-κB axis functions as an upstream trigger for innate immune activation and cytokine cascades in RA. Notably, TNF pathway modulation by TCM prescriptions may also mitigate altered autoantibody production associated with anti-TNF-α therapy (Alessandri et al., 2007), addressing a key unmet need in RA treatment.

Based on these findings and existing literature (Firestein, 2003; Taams, 2020; Ding et al., 2023), a shared inflammatory signaling axis—namely “TLR → NF-κB → TNF signaling/IL-17 signaling”—can be reasonably proposed. Integrating known RA inflammatory amplification mechanisms, this study formulates a testable “shared anti-inflammatory axis” hypothesis: the five prescriptions may exert consistent anti-inflammatory benefits by cooperatively suppressing the TLR4-MyD88-NF-κB-TNF/IL-17 signaling cascade, thereby reducing pro-inflammatory cytokine release and modulating immune cell differentiation. This shared regulatory scope further extends to bone metabolism-related pathways, where dysregulated Wnt signaling mediated by DKK1, Wnt5a, and β-catenin links inflammatory cascades to bone erosion (Riitano et al., 2025), supporting the holistic regulatory potential of TCM prescriptions in addressing both inflammation and structural damage in RA.

#### 4.3.3 Differential pathway enrichment and mechanistic biases among prescriptions

Beyond the shared inflammatory axis, each prescription exhibited distinct pathway enrichment preferences, providing mechanistically interpretable hypotheses for treatment differentiation under the same disease context.

Prescription 1 showed relatively strong enrichment in the AGE-RAGE signaling pathway and the Influenza A pathway (hsa05164), suggesting potential modulation of metabolic inflammation, oxidative stress, and infection-related immune activation loops. Previous studies indicate that RAGE and its ligands (e.g., AGEs, S100 proteins, HMGB1) contribute to RA synovial inflammation *via* NF-κB activation and interact with metabolic and oxidative stress-related inflammatory circuits. Additionally, IL-17 has been reported to promote RAGE expression in synovial cells, supporting biological crosstalk between the AGE-RAGE pathway and inflammatory axes (De Groot et al., 2011; Heo et al., 2011; Monu et al., 2022). Collectively, these findings suggest that Prescription 1 may be more relevant for RA patients with metabolic dysregulation or increased infection susceptibility, although this hypothesis requires validation through comorbidity-stratified and prospective studies.

Prescription 2 was uniquely enriched in the cytokine-cytokine receptor interaction pathway (hsa04060) and highly enriched in the Chagas disease pathway (hsa05142), indicating a potential bias toward broad cytokine network modulation and TLR2/4-related immune circuits. This profile suggests that Prescription 2 may be particularly relevant for RA subgroups characterized by heightened immune activation (e.g., high autoantibody titers). RA immunopathology indicates that cytokine networks (TNF-α, IL-6, IL-1, IL-17), innate immune receptors, and extracellular microvesicle-mediated dendritic cell activation (Buttari et al., 2025) jointly shape the synovial inflammatory microenvironment, providing a plausible explanation for the more pronounced network proximity observed for Prescription 2 (Firestein, 2003; Firestein and McInnes, 2017).

Prescription 3 exhibited prominent enrichment in IL-17 and TLR signaling pathways (odds ratio >120), alongside high enrichment in both the Chagas disease and AGE-RAGE pathways. These results support a hypothesis of coordinated suppression of inflammation-metabolism crosstalk. Mechanistically, Prescription 3 may attenuate neutrophil infiltration and tissue damage by modulating IL-17-TRAF6/ACT1-mediated expression of CXCLs, CSFs, and MMPs, while simultaneously inhibiting NF-κB and IRF3 activation through AGE-RAGE and TLR signaling interference. Given that IL-17, TLRs, and RAGE converge on shared transcriptional hubs such as NF-κB, this “multiple upstream receptors-shared downstream hubs” framework is mechanistically well supported (Firestein, 2003; De Groot et al., 2011; Heo et al., 2011; Taams, 2020; Monu et al., 2022; Ding et al., 2023). Accordingly, Prescription 3 may offer relative benefits for RA patients with active disease accompanied by metabolic abnormalities, pending validation through stratified clinical analyses.

Prescription 4 showed significant enrichment in osteoclast differentiation (hsa04380) and Th17 cell differentiation pathways (hsa04659), suggesting a potential advantage in dual regulation of bone destruction and immune inflammation. By modulating the RANKL/RANK axis—coordinated with the regulation of Wnt signaling pathway key molecules (DKK1, Wnt5a, and β-catenin) (Riitano et al., 2025)—this prescription may inhibit NF-κB and NFATc1 activation to slow bone erosion, while concurrently influencing Th17/Treg balance *via* STAT3/RORγt and STAT5/Foxp3 pathways. Given that RANKL-driven osteoclastogenesis and Th17-mediated inflammation form a critical coupling mechanism in RA structural damage (Taams, 2020), Prescription 4 may hold particular potential for RA patients at higher risk of bone erosion. Molecular docking further demonstrated strong binding affinity between berberine and PTGS2 (−10.210 kcal/mol), providing structural support for its potential analgesic and anti-inflammatory effects. Previous studies reporting the anti-inflammatory and immunomodulatory activities of berberine further corroborate this finding (Schett and Gravallese, 2012; Taams, 2020; Sun et al., 2024). This anti-erosive potential may involve modulation of the Wnt signaling pathway, given its central role in regulating osteoblast-osteoclast coupling and its well-documented dysregulation in RA-related bone loss.

Prescription 5 was highly enriched in the Fluid shear stress and atherosclerosis pathway (hsa05418) and uniquely enriched in the inflammatory bowel disease pathway (hsa05321). Gene Ontology analysis also highlighted enrichment in responses to lipopolysaccharide, suggesting a regulatory bias toward endothelial function and mucosal/gut immune inflammation, and autophagy-mediated post-translational modifications of proteins and extracellular vesicle release (Riitano et al., 2023). Given the increased cardiovascular risk associated with RA and emerging evidence linking gut dysbiosis and mucosal immune activation to RA pathogenesis *via* the gut-joint axis (Wang et al., 2022; Tsetseri et al., 2023; Pamies et al., 2025), Prescription 5 may confer potential benefits in RA patients with cardiovascular metabolic comorbidities or intestinal immune imbalance, although further stratified investigations are required.

#### 4.3.4 Molecular docking as structural support for network predictions

Following system-level inference, molecular docking analyses were conducted using the core RA targets TNF-α and PTGS2 to evaluate the structural plausibility of direct compound-target interactions. Docking results indicated that multiple natural compounds across prescriptions demonstrated favorable predicted binding affinities toward TNF-α and PTGS2, with binding energies generally below −7.0 kcal/mol, suggesting potential compatibility within the binding pockets. For instance, quercetin from Prescription 1 exhibited a predicted binding energy of −8.632 kcal/mol with TNF-α, while kaempferol showed a binding energy of −9.664 kcal/mol with PTGS2. Berberine from Prescription 4 displayed a comparatively lower predicted binding energy with PTGS2 (−10.210 kcal/mol), indicating a potentially stable interaction at the structural level. These findings provide supportive structural evidence for a multi-component, multi-target interaction framework, although experimental validation would be required to confirm biological activity.

While these docking results provide encouraging structural plausibility for interactions between our prioritized compounds and key RA targets, several important caveats must be explicitly acknowledged regarding the compound selection strategy. First, the selection was inherently biased towards compounds with high predicted oral bioavailability (OB ≥ 30%) and drug-likeness (DL ≥ 0.18), as defined by the TCMSP database. This filter, while useful for narrowing down candidates, may inadvertently exclude compounds that act as prodrugs, are metabolized into active forms *in vivo*, or exert synergistic effects through modulation of gut microbiota or host metabolism. Second, the prioritization of compounds with well-documented anti-inflammatory activities (e.g., quercetin, berberine) introduces a confirmation bias, potentially overlooking novel or less-characterized constituents that may contribute to the overall therapeutic effects. Third, given the vast chemical diversity of multi-herb prescriptions, the number of compounds assessed in this docking study represents only a small fraction of the total chemical space. Therefore, the strong binding affinities observed for compounds like quercetin and berberine should be interpreted as proof-of-concept evidence that the molecular network associations predicted by network pharmacology are structurally feasible for at least some of the prescription’s constituents. They are not intended to suggest that these are the exclusive, or even the primary, active ingredients responsible for the clinical efficacy observed in the CRP analysis.

Given these inherent limitations in compound selection, it should be emphasized that molecular docking primarily evaluates the structural feasibility and binding plausibility of compound-target interactions at the computational level. Docking scores and binding energies should therefore be interpreted with caution, as they do not directly reflect biological potency or *in vivo* efficacy. The present docking analysis mainly serves as a complementary computational approach to support network pharmacology predictions and generate testable hypotheses (Cui and Jia, 2021; Shen et al., 2021; Tang et al., 2022; Gao et al., 2024; Li et al., 2024). Further experimental validation using biochemical assays, cellular experiments, or animal models is required to confirm the functional relevance of these predicted interactions.

### 4.4 Innovations and limitations

The main contributions of this study include: (1) extraction of interpretable and reproducible core prescriptions from highly heterogeneous real-world data using frequent closed itemset compression and difference-ratio screening; (2) provision of directional clinical efficacy signals *via* CRP changes in patients with ≥80% prescription coverage; (3) integration of network pharmacology, network proximity metrics, and molecular docking to generate testable hypotheses for shared and prescription-specific mechanisms, thereby supporting prescription-phenotype matching concepts.

Nevertheless, several limitations should be acknowledged. First, as a retrospective real-world study, the findings are subject to baseline heterogeneity, concomitant medications, and confounding factors, and should therefore be interpreted as hypothesis-generating rather than definitive evidence (Schneeweiss, 2014; Sherman et al., 2016; Corrigan-Curay et al., 2018). Second, network pharmacology and enrichment analyses provide associative rather than causal inference, and molecular docking represents computational simulation. Furthermore, the compound selection strategy for docking was inherently biased towards well-studied, database-predicted active compounds (e.g., those meeting OB ≥ 30% and DL ≥ 0.18 filters). This approach, while practical for initial exploration, may not capture the full pharmacological complexity of the prescriptions, potentially overlooking prodrugs, metabolites, or synergistic compound combinations that contribute to the overall therapeutic effect. Consequently, the docking findings should be interpreted as exploratory and hypothesis-generating rather than a comprehensive characterization of the prescriptions’ active principles. Accordingly, the mechanistic predictions derived from network pharmacology and molecular docking require further validation through *in vitro* and *in vivo* experimental studies to substantiate compound-target interactions and pathway-level effects. Third, the present study relied primarily on longitudinal CRP changes as the available objective clinical indicator. Standardized composite RA activity measures, such as DAS28, as well as structured joint counts and imaging-based structural assessments, were not systematically recorded in the retrospective EMR systems of the two hospitals. Although ESR is another commonly used inflammatory marker, its testing frequency was relatively low and inconsistent among patients, leading to substantial missing data that precluded robust statistical analysis. Consequently, comprehensive evaluation of disease activity states (e.g., remission, low disease activity) and structural progression could not be performed, nor could treat-to-target-based response assessments be formally conducted. These data limitations reflect the characteristics of real-world inpatient clinical practice rather than study design constraints, and therefore the efficacy findings should be interpreted cautiously and considered exploratory rather than confirmatory. Fourth, although network pharmacology analyses suggested prescription-specific pathway enrichment patterns, the relevance of these mechanistic differences to distinct clinical phenotypes or patient subgroups could not be formally validated within the current dataset. The retrospective EMR data did not contain sufficiently detailed stratification variables—such as baseline disease activity categories, serological status (e.g., RF/ACPA), imaging severity, or comorbidity-defined subgroups—to enable subgroup-level comparative analysis. Therefore, the proposed prescription-phenotype matching hypotheses remain inferential and require validation through prospective, stratified, or multicenter studies incorporating standardized clinical outcomes and predefined subgroup analyses.

## 5 Conclusion

Within an integrated evidence-chain framework encompassing data mining, clinical efficacy validation, network pharmacology, and molecular docking, this study analyzed 4,729 RA prescriptions derived from inpatient electronic medical records from two tertiary hospitals between November 2019 and August 2023. Using frequent closed itemset compression, representative core prescriptions were extracted, systematically characterizing shared and distinct medication patterns used by different physicians in RA treatment.

At the real-world clinical level, all five core prescriptions were associated with significant post-treatment reductions in CRP, indicating stable anti-inflammatory signals at the biomarker level and providing a clinically contextualized framework for subsequent mechanistic hypothesis generation. Mechanistically, network pharmacology analyses revealed substantial overlap between prescription targets and RA disease targets (65–115 shared targets) and convergent enrichment in key inflammatory and immune pathways, including Toll-like receptor, IL-17, and TNF signaling. Network proximity metrics further supported strong topological associations between prescription targets and the RA disease module. At the structural level, molecular docking simulations provided computational evidence for the structural feasibility of interactions between representative active compounds (e.g., quercetin and berberine) and core RA-related targets such as TNF-α and PTGS2, offering structural plausibility rather than experimental confirmation of biological activity. Collectively, these multi-level findings generate mechanistic hypotheses that warrant further *in vitro* and *in vivo* validation.

In summary, by integrating real-world clinical data with systems pharmacology and structural biology approaches, this study suggests that although different TCM schools apply distinct prescriptions in RA treatment, these prescriptions converge on shared inflammatory and immune axes while retaining prescription-specific emphases. These findings provide computational support for the multi-component, multi-target characteristics of traditional Chinese medicine in this RA context and provide a methodological and evidentiary foundation for refining RA medication patterns and advancing future prescription-phenotype matching research.

## Data Availability

The underlying datasets supporting the findings of this study’s specifically, the raw electronic medical records from the First Affiliated Hospital of Henan University of Chinese Medicine and Wangjing Hospital of the China Academy of Chinese Medical Sciences’ are subject to strict restrictions in order to protect patient privacy and comply with relevant regulations and ethical approval requirements. Requests to access these datasets should be directed to LZ (E-mail: tcmxpzl@126.com).
