# Supplementary material for: Mining personalized core traditional Chinese medicine prescriptions for rheumatoid arthritis and elucidating their mechanisms via frequent closed Itemset compression and multilevel network pharmacology
Source: Front Mol Biosci. 2026 Mar 23;13:1792988. doi: 10.3389/fmolb.2026.1792988 (PMC13051219; doi:10.3389/fmolb.2026.1792988)
Supplement: Supplementary file 1 [file Table1.docx]

Supplementary Table S1. Herbal Nomenclature: Pinyin, English, and Latin Names

| **Pinyin** | **Pharmacopoeia Name** | **Latin Botanical Name** |
| --- | --- | --- |
| AiYe | Artemisiae Argyi Folium | Artemisia argyi Lévl. & Vaniot |
| BaiShao | Paeoniae Radix Alba | Paeonia lactiflora Pall. |
| BaiZhi | Angelicae Dahuricae Radix | Angelica dahurica (Hoffm.) Benth. & Hook.f. |
| BaiZhu | Atractylodis Macrocephalae Rhizoma | Atractylodes macrocephala Koidz. |
| BiXie | Dioscoreae Hypoglaucae Rhizoma | Dioscorea hypoglauca Palib. |
| BieJia | Trionycis Carapax | Pelodiscus sinensis (Wiegmann) |
| CaoWu | Aconiti Kusnezoffii Radix Cocta | Aconitum kusnezoffii Reichb. |
| CheQianZi | Plantaginis Semen | Plantago asiatica L. |
| ChenPi | Citri Reticulatae Pericarpium | Citrus reticulata Blanco |
| ChiShao | Paeoniae Radix Rubra | Paeonia veitchii Lynch |
| ChuanNiuXi | Cyathulae Radix | Cyathula officinalis K.C. Kuan |
| ChuanShanLong | Dioscoreae Nipponicae Rhizoma | Dioscorea nipponica Makino |
| ChuanWu | Aconiti Radix Cocta | Aconitum carmichaelii Debeaux |
| ChuanXiong | Chuanxiong Rhizoma | Ligusticum chuanxiong Hort. |
| DaHuang | Rhei Radix et Rhizoma | Rheum palmatum L. |
| DanShen | Salviae Miltiorrhizae Radix et Rhizoma | Salvia miltiorrhiza Bunge |
| DangGui | Angelicae Sinensis Radix | Angelica sinensis (Oliv.) Diels |
| DangShen | Codonopsis Radix | Codonopsis pilosula (Franch.) Nannf. |
| DaXueTeng | Sargentodoxae Caulis | Sargentodoxa cuneata (Oliv.) Rehder & E.H. Wilson |
| DaZao | Jujubae Fructus | Ziziphus jujuba Mill. |
| DanZhuYe | Lophatheri Herba | Lophatherum gracile Brongn. |
| DiFengPi | Periplocae Cortex | Periploca sepium Bunge |
| DiGuPi | Lycii Cortex | Lycium chinense Mill. |
| DuHuo | Angelicae Pubescentis Radix | Angelica pubescens Maxim. |
| DuYiWei | Lamiophlomis Herba | Lamiophlomis rotata (Benth.) Kudo |
| DuZhong | Eucommiae Cortex | Eucommia ulmoides Oliv. |
| FangFeng | Saposhnikoviae Radix | Saposhnikovia divaricata (Turcz.) Schischk. |
| FengXianTouGuCao | Impatientis Herba | Impatiens balsamina L. |
| FoShou | Citri Sarcodactylis Fructus | Citrus medica var. sarcodactylis Swingle |
| FuLing | Poria | Poria cocos (Schw.) Wolf |
| GanCao | Glycyrrhizae Radix et Rhizoma | Glycyrrhiza uralensis Fisch. |
| GanJiang | Zingiberis Rhizoma | Zingiber officinale Roscoe |
| GeGen | Puerariae Lobatae Radix | Pueraria lobata (Willd.) Ohwi |
| GuiJianYu | Sargentodoxae Caulis | Sargentodoxa cuneata (Oliv.) Rehder & E.H. Wilson |
| GuiZhi | Cinnamomi Ramulus | Cinnamomum cassia (L.) J. Presl |
| GuSuiBu | Drynariae Rhizoma | Drynaria fortunei (Kuntze) J. Sm. |
| HongHua | Carthami Flos | Carthamus tinctorius L. |
| HuangBai | Phellodendri Cortex | Phellodendron amurense Rupr. |
| HuangQi | Astragali Radix | Astragalus membranaceus (Fisch.) Bunge |
| HuZhang | Polygoni Cuspidati Rhizoma et Radix | Polygonum cuspidatum Siebold & Zucc. |
| JiNeiJin | Galli Gigerii Endothelium Corneum | Gallus gallus domesticus Brisson |
| JiangCan | Bombyx Batryticatus | Bombyx mori L. |
| JinYinTeng | Lonicerae Caulis | Lonicera japonica Thunb. |
| KeTengZi | Entadae Semen | Entada phaseoloides (L.) Merr. |
| LiuJiNu | Artemisiae Anomalae Herba | Artemisia anomala S. Moore |
| LongYanRou | Longan Arillus | Dimocarpus longan Lour. |
| LuLuTong | Liquidambaris Fructus | Liquidambar formosana Hance |
| MaHuang | Ephedrae Herba | Ephedra sinica Stapf |
| MaiYa | Hordei Fructus Germinatus | Hordeum vulgare L. |
| MangXiao | Natrii Sulfas | Sodium sulfate decahydrate |
| MiHouTaoGen | Actinidiae Radix | Actinidia chinensis Planch. |
| MoHanLian | Ecliptae Herba | Eclipta prostrata (L.) L. |
| MoYao | Myrrha | Commiphora myrrha (Nees) Engl. |
| MuDanPi | Moutan Cortex | Paeonia suffruticosa Andrews |
| MuGua | Chaenomelis Fructus | Chaenomeles speciosa (Sweet) Nakai |
| MuXiang | Aucklandiae Radix | Aucklandia lappa Decne. |
| NanFangHongDouShan | Taxus Cuspidatae Folium et Ramulus | Taxus chinensis var. mairei (Lemée & H. Lév.) Cheng & L.K. Fu |
| NiuXi | Achyranthis Bidentatae Radix | Achyranthes bidentata Blume |
| NvZhenZi | Ligustri Lucidi Fructus | Ligustrum lucidum W.T. Aiton |
| PuGongYing | Taraxaci Herba | Taraxacum mongolicum Hand.-Mazz. |
| QianNianJian | Homalomenae Rhizoma | Homalomena occulta (Lour.) Schott |
| QiangHuo | Notopterygii Rhizoma et Radix | Notopterygium incisum Ting ex H.T. Chang |
| QinPi | Fraxini Cortex | Fraxinus chinensis Roxb. |
| QingFengTeng | Sinomenii Caulis | Sinomenium acutum (Thunb.) Rehder & E.H. Wilson |
| QingHao | Artemisiae Annuae Herba | Artemisia annua L. |
| RenDongTeng | Lonicerae Caulis | Lonicera japonica Thunb. |
| RuXiang | Olibanum | Boswellia carterii Birdw. |
| SangJiSheng | Taxilli Herba | Taxillus chinensis (DC.) Danser |
| ShaRen | Amomi Fructus | Amomum villosum Lour. |
| ShenJinCao | Lycopodii Herba | Lycopodium japonicum Thunb. |
| ShengDiHuang | Rehmanniae Radix | Rehmannia glutinosa (Gaertn.) DC. |
| ShengJiang | Zingiberis Rhizoma Recens | Zingiber officinale Roscoe |
| ShenQu | Massa Medicata Fermentata | Fermented mixture |
| SuMu | Sappan Lignum | Caesalpinia sappan L. |
| SuanZaoRen | Ziziphi Spinosae Semen | Ziziphus jujuba var. spinosa (Bunge) Hu ex H.F. Chow |
| TaiZiShen | Pseudostellariae Radix | Pseudostellaria heterophylla (Miq.) Pax |
| TaoRen | Persicae Semen | Prunus persica (L.) Batsch |
| TianNanXing | Arisaematis Rhizoma | Arisaema erubescens (Wall.) Schott |
| TuFuLing | Smilacis Glabrae Rhizoma | Smilax glabra Roxb. |
| WeiLingXian | Clematidis Radix et Rhizoma | Clematis chinensis Osbeck |
| XiXin | Asari Radix et Rhizoma | Asarum heterotropoides F. Schmidt |
| XianMao | Curculiginis Rhizoma | Curculigo orchioides Gaertn. |
| XunGuFeng | Sarcopyramidis Herba | Sarcopyramis nepalensis Wall. |
| YanHuSuo | Corydalis Rhizoma | Corydalis yanhusuo W.T. Wang |
| YiYiRen | Coicis Semen | Coix lacryma-jobi L. |
| YinChaiHu | Stellariae Radix | Stellaria dichotoma L. |
| YinYangHuo | Epimedii Folium | Epimedium brevicornum Maxim. |
| ZeXie | Alismatis Rhizoma | Alisma orientale (Sam.) Juzep. |
| ZhenZhuTouGuCao | Speranskiae Herba | Speranskia tuberculata (Bunge) Baill. |
| ZhiGanCao | Glycyrrhizae Radix et Rhizoma Praeparata cum Melle | Glycyrrhiza uralensis Fisch. |
| ZhiMu | Anemarrhenae Rhizoma | Anemarrhena asphodeloides Bunge |
| ZhiQiao | Aurantii Fructus | Citrus aurantium L. |
| ZhiYuanZhi | Polygalae Radix Praeparata | Polygala tenuifolia Willd. |
